# Methionine sources at different dietary levels alters the growth and expression of genes related to homocysteine remethylation in the jejunum of broilers

**DOI:** 10.1371/journal.pone.0291998

**Published:** 2023-11-13

**Authors:** Tamires M. S. Felix, Claudiana S. Souza, Silvana C. L. Santos, Danila B. Campos, Samuel E. Aggrey, Ricardo R. Guerra, José H. V. Silva

**Affiliations:** 1 Departament of Animal Science, Center of Agrarian Sciences, Universidade Federal da Paraíba, Areia, Paraíba, Brazil; 2 Departament of Agriculture, Center for Human, Social and Agricultural Sciences, Universidade Federal da Paraíba, Bananeiras, Paraíba, Brazil; 3 Departament of Veterinary Sciences, Center of Agrarian Sciences, Universidade Federal da Paraíba, Areia, Paraíba, Brazil; 4 NutriGenomics Laboratory, Department of Poultry Science, University of Georgia, Athens, Georgia, United States of America; University of Illinois, UNITED STATES

## Abstract

Sulfur amino acids are essential for the proper development of broilers and are required throughout the bird’s life to perform important physiological functions. Studies that seek to understand the actions of sulfur amino acids in the body of birds are essential. The present study evaluated the influence of sulfur amino acid supplementation using DL-Methionine (DL-Met) and DL-Methionine hydroxy analogue (DL-HMTBA), on the performance and expression of genes related to methionine metabolism, in the jejunum of broilers. Four hundred and fifty male broilers (Cobb-700 slow feathering) were distributed in a completely randomized design, in a factorial scheme (2x3), with two sources of methionine (DL-Met and DL-HMTBA) and three levels of methionine (deficiency, requirement and excess). The mRNA expression of the MAT1, MTR, BHMT, MTRR, CBG and GSS genes, and performance data such as feed intake, weight gain, and feed conversion were evaluated. DL-HMTBA increased the expression of BHMT (p = 0.0072) and MTRR (p = 0.0003) in the jejunum of the birds. Methionine deficiency increased the expression of BHMT (p = 0.0805) and MTRR (p = 0.0018). Higher expression of GSS was observed in birds that were supplemented with DL-HMTBA (p = 0.0672). Analyzing our results, it is preferable to supplement sulfur amino acids with DL-Met at the requirement level. Birds fed with DL-HMTBA showed worse weight gain (p = 0.0117) and higher feed conversion (p = 0.0170); methionine deficiency resulted in higher feed intake (p = 0.0214), lower weight gain (p<0.0001) and consequently higher feed conversion (p<0.0001). Based on the information found in this work, it is recommended to supplement sulfur amino acids with DL-Met at the level of compliance with the requirement.

## Introduction

Sulfur amino acids are essential for the growth and development of broilers throughout the bird’s life and are therefore widely studied. Methionine performs important physiological functions in the body of birds and is a methyl group donor for methylation reactions of important molecules, such as DNA [1]. It is also used in the production of glutathione peroxidase (GSH-Px), a key enzyme in enzymatic antioxidant action against free radicals [2].

Methionine supplementation in broilers increases the expression of genes related to cysteine, GSH and GSH-Px synthesis, mitigating the effects of oxidative stress [3]. Moreover, methionine is also related to the synthesis of polyamines (spermidine and spermine), which are associated with cell division, growth, and proliferation [4].

To perform bioactive functions in the organism, methionine has a complex metabolism, going through several biochemical reactions that can be divided into three stages: methylation, remethylation and transsulfuration. In methylation, the conversion of methionine to S-adenosylmethionine (SAM) occurs. This reaction is catalyzed by methionine adenosyltransferases (MAT) enzymes, and then SAM is converted into S-adenosyl-homocysteine (SAH) [5].

SAH can follow two pathways. It can be converted to homocysteine through the transsulfuration pathway, with the action of cystathione-β-synthetase (CBS) enzyme, and this reaction is irreversible [6]. Or remethylation occurs, in which homocysteine is regenerated into methionine, with the action of methionine synthase (MS) enzyme and a donor of a methyl group, which can come in two ways [7].

The choline/betaine pathway, which requires the action of the betaine homocysteine methyltransferase (BHMT) enzyme, or the tetrahydrofolate pathway, which requires the action of the 5-methyltetrahydrofolate-homocysteine methyltransferase (MTR) and 5-methyltetrahydrofolate-homocysteine methyltransferase reductase (MTRR) enzymes [5].

Vegetable sources are poor in methionine, and to meet the requirements of sulphurous amino acids, it is necessary to supplement them via diet. DL-Methionine (DL-Met; 98% methionine), in powder form, and its analogue, DL-Methionine Hydroxy Analogue (DL-HMTBA; 88% methionine), in liquid form, are the most common sources used for supplementation [2].

These sources of methionine are absorbed and used in different ways by birds, which are able to use only the L-form of amino acids, so D-Met and both DL-HMTBA isomers need to be converted to L-Met, with the action of deaminase and transaminase enzymes [2]. These enzymes are found in greater amounts in the liver and kidneys, and in smaller amounts in the small intestine, to support the splanchnic metabolism [8]. L-Met is transported in the jejunum of chickens by four transport systems, which transport the intestinal lumen into the enterocyte [9].

Due to the importance of sulfur amino acids for the proper functioning of the body of birds, it is important to carry out studies that evaluate the metabolism of these amino acids. Thus, the aim of this study was to investigate the effects of sulfur amino acid supplementation on the performance and expression of genes related to methionine metabolism, in the jejunum of broilers, by using two sources of methionine and at different nutritional levels.

## Material and methods

### Management and housing

The experimental protocols were carried out in accordance with the precepts and norms issued by the National Council for the Control of Animal Experimentation (CONCEA), having been approved by the Ethics Commission for the Use of Animals at the Federal University of Paraíba (CEUA-UFPB), under the protocol number 075/2017.

The experimental period was 42 days and used 450 one-day-old Cobb-700 lineage (slow feathering) male broilers. The birds were purchased from a commercial hatchery and vaccinated against Marek and Gumboro diseases. Until the 7th day of age, the chicks were kept in protection circles. On the eighth day of age, they were weighed and divided into groups of 15 birds (0.185 + 0.001 kg of weight) per experimental unit in stalls with an area of 1.5 m^2^, equipped with tube feeders and pendulum drinkers, with an *ad libitum* supply of water and feed.

Wood shavings were used to cover the floor of the stalls. The birds were heated with infrared lamps, keeping the levels of temperature and relative humidity in the thermoneutral range according to the age of the birds. The lighting program was 18 hours of light (natural + artificial) and 6 hours of darkness.

### Animals, design, and experimental diets

The experiment was carried out in a completely randomized design, in a 2x3 factorial scheme, with two sources (DL-Met and DL-HMTBA) and three levels of Met+CysD, one of which was deficient, with a 0.25 reduction in the requirement, the other level considered in the requirement (LysD:Met+CysD ratio 0.72 and 0.73 to starter, and grower/finisher, respectively), and the last level with an excess of 0.25 of the requirement, totaling six treatments, each with five repetitions of 15 birds. There were three phases in the experiment—starter (8 to 21 days), grower (21 to 33 days), and finisher (34 to 42 days). Three reference diets were formulated for each phase of the study according to the recommendations of [10], meeting the requirements of the birds, except for the levels of Met+CysD, which were below what is recommended (Table 1).

**Table 1:** Feed composition and nutrient contents of diets formulated for broilers.

Ingredients, %	DL-Methionine			DL-HMTBA		
	Starter (8-21d)	Grower (22-33d)	Finisher (34-42d)	Starter (8-21d)	Grower (22-33d)	Finisher (34-42d)
Corn (7.88%)	56.716	62.175	62.506	56.716	62.175	62.506
Soybean meal (45%)	35.775	30.132	28.685	35.775	30.132	28.685
Soy oil	3.291	3.692	4.500	3.291	3.692	4.500
Dicalcium phosphate (18.1%)	1.558	1.353	1.151	1.558	1.353	1.151
Limestone	0.945	0.889	0.759	0.945	0.889	0.759
DL-Methionine (98%)	0.062	0.060	0.014	-	-	-
DL-HMTBA (88%)	-	-	-	0.070	0.068	0.016
L-Lysine HCl (78.4%)	0.245	0.308	0.315	0.245	0.308	0.315
L-Threonine (99%)	0.084	0.103	0.144	0.084	0.103	0.144
L-Arginine (99%)	0.000	0.058	0.191	0.000	0.058	0.191
L-Valine (99%)	0.000	0.086	0.084	0.000	0.086	0.084
L-Tryptophan (99%)	0.000	0.000	0.024	0.000	0.000	0.024
Starch	0.400	0.400	0.400	0.400	0.400	0.400
Salt	0.491	0.338	0.743	0.491	0.338	0.743
Choline chloride (60%)	0.100	0.070	0.100	0.100	0.070	0.100
Vitamin premix^1^	0.050	0.050	0.100	0.050	0.050	0.100
Mineral premix^2^	0.050	0.050	0.050	0.050	0.050	0.050
Coccidiostat^3^	0.005	0.005	0.005	0.005	0.005	0.005
Bacitracin zinc	0.015	0.015	0.015	0.015	0.015	0.015
Antioxidant^4^	0.010	0.010	0.010	0.010	0.010	0.010
Inert^5^	0.200	0.200	0.200	0.200	0.200	0.200
Total	100.00	100.00	100.00	100.00	100.00	100.00
Calculated energy and nutrients content, %						
ME (kcal/kg)	3050.00	3150.00	3200.00	3050.00	3150.00	3200.00
Crude protein	20.98	19.00	18.40	20.98	19.00	18.40
Calcium	0.841	0.758	0.663	0.841	0.758	0.663
Avalilable phosphor	0.401	0.354	0.309	0.401	0.354	0.309
Methionine	0.343	0.318	0.266	0.343	0.318	0.266
Methionine+Cystine	0.630	0.580	0.520	0.630	0.580	0.520
Threonine	0.791	0.735	0.750	0.791	0.735	0.750
Tryptofan	0.236	0.206	0.220	0.236	0.206	0.220
Lysine	1.217	1.131	1.100	1.217	1.131	1.100
Valine	0.891	0.880	0.850	0.891	0.880	0.850
Arginine	1.327	1.221	1.300	1.327	1.221	1.300
Sodium	0.210	0.150	0.310	0.210	0.150	0.310
Chlorine	0.345	0.254	0.495	0.345	0.254	0.495
Potassium	0.819	0.732	0.706	0.819	0.732	0.706

Mogin number (mEq/kg): Starter: 203.59; Grower: 180.63; Finisher: 176.07.

^1^Vitamin premix: Vit A 1.000.000 UI; Vit D3 1.700.000 UI; Vit E 20.000 mg; Vit K3 2.000 mg; Vit B1 2.000 mg; Vit B2 4.000 mg; Vit B6 2.000 mg; Vit B12 10.000 mcg; Niacin 20.000 mg; Pantothenate acid 10.000 mg; Biotin 25 mg; Folic acid 500 mg;

^2^Mineral premix: Selenium 250 mg; Manganese 75.000 mg; Zinc 70.000 mg; Iron 50.000 mg; Copper 8.500 mg; Iodine 1.500 mg; Cobalt 200 mg.

^3^Salinomycin sodium salt 12g.

^4^Butylated hydroxytoluene (BHT) 200 ppm.

^5^Kaolin.

The reference diets in each phase were supplemented to meet the requirement or excess of Met+CysD with DL-Met (98%) or DL-HMTBA (88%), the latter source was added on an equimolar basis of DL-Met, both replacing the inert. The diets were isonitrogenous and isoenergetic. Equimolar substitution levels of DL-Met for DL-MHA-FA were 0 and 100%. Experimental dietary levels are shown in Table 2.

**Table 2:** Experimental methionine levels.

Phase	Levels	DL-Methionine (98%)		DL-HMTBA (88%)	
		%Met+Cys diet	%Addition level	%Met+Cys diet	%Addition level
Starter (8-21d)	Less 0.25	0.63	0.062	0.63	0.070
	Requirement	0.88	0.317	0.88	0.360
	Plus 0.25	1.13	0.572	1.13	0.645
Grower (22-33d)	Less 0.25	0.58	0.060	0.58	0.068
	Requirement	0.83	0.315	0.83	0.354
	Plus 0.25	1.08	0.570	1.08	0.641
Finisher (34-42d)	Less 0.25	0.52	0.014	0.52	0.016
	Requirement	0.77	0.269	0.77	0.303
	Plus 0.25	1.02	0.524	1.02	0.590

### mRNA isolation, cDNA preparation, and real time PCR analysis

At 43 days of the experiment, five birds were randomly selected per treatment and subjected to solid fasting for 12 hours for complete emptying of the digestive tract, then the birds were stunned by electrocution and slaughtered, the jejunum samples were collected, in order to investigate methionine metabolism genes. The samples were macerated in liquid nitrogen, the total mRNA was extracted by using the Trizol^®^ reagent (Invitrogen, Carlsbad CA, USA), purified with the RNeasy Mini Kit (Qiagen, Valencia CA, number 74.104) and treated with an RNase inhibitor, of according to the manufacturers’ recommendations. The RNA was suspended in water treated with diethyl pyrocarbonate (DEPC water, Sigma Aldrich^®^). The RNA concentration and purity were determined by using the absorbance ratios of 260/280 and 260/230 in a spectrophotometer (Colibri), and they were stored at -80°C.

A total of 2μg of mRNA was reverse transcribed with the High-Capacity cDNA RT kit (Thermo Fischer, number 4368814) following the kit’s standard analytical protocol, which recommends the following conditions: 25ºC for 10 minutes, 37ºC for 120 minutes, 85ºC for 5 minutes and 4ºC, for a total of 4 hours. These conditions were performed in a conventional polymerase chain reaction (PCR) thermocycler. Then, 1μL of cDNA was taken as a standard template in a 20μl qPCR mix containing 1μl of each primer (forward and reverse from 10μM) and 2X Fast SYBR Green Master Mix (Agilent Technologies^®^).

Real-time polymerase chain reaction (RT-PCR) cycles were performed on a Stragene mx3000p thermal cycler (Agilent Technologies^®^), at 95°C for 20 seconds, followed by 40 cycles of 95°C for 3 seconds and 60°C for 30 seconds and 95ºC for 15 seconds, 60ºC for 1 minute and finally, 95ºC for 15 seconds. Furthermore, at the end of each reaction, the melting temperature curve of each RT-PCR reaction was determined.

RT-PCR analyses of each of the 5 samples were performed in triplicates. Relative gene expression was calculated based on the 2-ΔΔCt method [11] and was normalized by the expression of the β-actin gene in each sample, as reference gene. GenBank accession numbers, forward and reverse primers of the genes used in the study are available in Table 3.

**Table 3:** Primers used for quantitative real time PCR in *Gallus gallus domesticus*.

Gene symbol^1^	GenBank ID	Description/Function	Forward/Reverse primer
MAT1	NM_001199519.2	Catalyzes the transfer of adenosine from ATP to methionine, forming SAM.	TCATACCAGTGCGTGTCCAT CACACGATCCTTCAGGGTTT
MTR	XM_046914306.1	Catalyzes the final step in methionine biosynthesis from homocysteine.	TACACCGGCACATATCAGGA CCAGACCTGACAGCAGCATA
BHMT	XM_414685.7	Catalyzes the transfer of a methyl group from trimethylglycine to produce methionine.	GGTGCTTCCATTGTTGGAGT CAGGTGGGCTTTCAGCTTAG
MTRR	XM_004935129.5	Regenerates MTR to a functional state.	ATTGATGGTCTTTGGCTTGC AACATGTGGGTCTGCACTGA
CBS	XM_416752.7	Catalyzes the first step of the transsulfuration pathway, from homocysteine to cystathionine.	CTGGGATCTTGAAACCTGGA ACAGCGGTAACCCTTCACTG
GSS	XM_425692.8	Catalyzes the reaction of cysteine to glutathione.	TTGCTGGGCTGTACTCACTG CTCCTTCTCGCTGTGGTTTC
β-Actin	L_08165.1	-	ACCCCAAAGCCAACAGA CCAGAGTCCATCACAATACC

^1^MAT1: methionine adenosyltransferase 1; MTR: 5 methyltetrahydrofolate-homocysteine methyltransferase; BHMT: betaine-homocysteine S-methyltransferase; MTRR: 5-methyltetrahydrofolate-homocysteine methyltransferase reductase; CBS: cystathionine beta synthase; GSS: gluthathione synthetase.

### Performance

The feed intake and weight gain of the birds were measured in each experimental plot. Weighing occurred at the beginning and end of the experiment (8 to 42 days). Feed conversion was calculated by using the amount of feed consumed by the chickens in each plot divided by the weight gain.

### Statistical analysis

Normality assumptions were verified by using the Shapiro-Wilk test. Performance and gene expression data were submitted to analysis of variance, according to the statistical model below:

$$\text{Yij}\left(\text{k}\right)=\mu +\text{Si}+\text{Nj}+\text{SxNij}+\text{eij}\left(\text{k}\right)$$

Where, Yij(k) = value observed for the variable studied; μ = general average; Si = i-th effect of methionine source; Nj = effect of the j-th of methionine levels; SxNij = effect of the interaction between methionine sources and methionine levels and eij = experimental error.

After the ANOVA test, the gene expression data were submitted to the t test. The differences were significant when p≤0.10. The performance data were submitted to the Tukey test and the differences were significant when p≤0.05. Statistical analyses were performed by using the SAS PROC GLM (Version 9.1, SAS Inst. Inc., Cary, NC).

## Results

**Fig 1:**
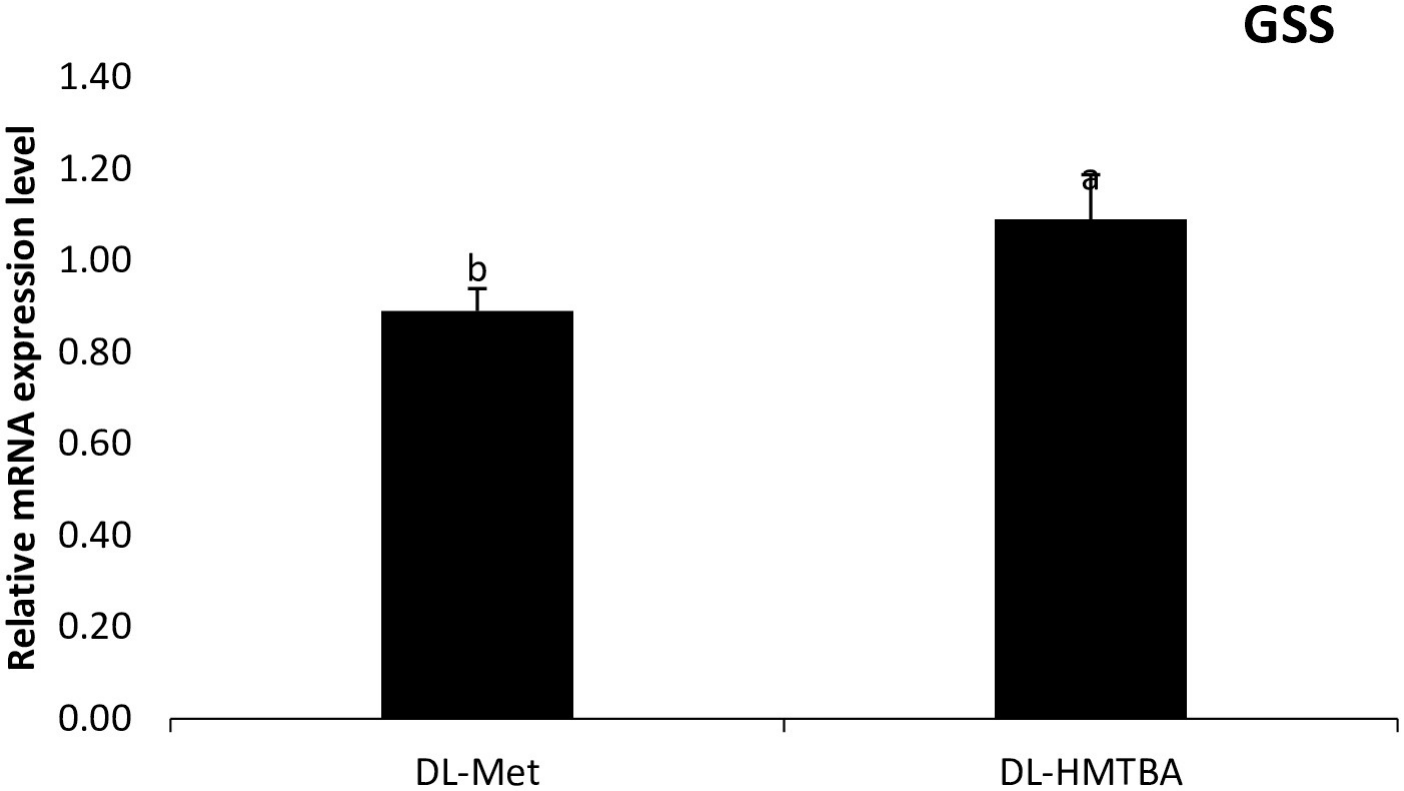
Graphic representation of the mRNA expression of gluthathione synthetase (GSS) in the jejunum of broilers (mean±SEM; n = 6). ^a-b^Different letters indicate a statistical difference by test t with p<0.10.

Gene expression data are shown on Table 4. Analyzing the effects of the sources used in this study, on the expression of methionine metabolism genes in the jejunum of broilers, DL-HMTBA increased the gene expression of BHMT (p = 0.0072), MTRR (p = 0.0003), and GSS (p = 0.0672) (Fig 1).

**Table 4:** Effect of increasing levels of methionine+cystine and replacement of DL-Met by DL-HMTBA in the diet on gene expression of genes of methionine metabolism of broilers from 8 to 42 days of age.

Main effects^1^	MAT1	MTR	BHMT	MTRR	CBS	GSS
Source						
DL-Met	0.743±0.082	0.985±0.068	0.708±0.094^b^	0.733±0.040^b^	0.977±0.116	0.892±0.048^b^
DL-HMTBA	0.706±0.080	0.930±0.138	1.087±0.114^a^	1.313±0.186^a^	1.118±0.091	1.091±0.097^a^
*P value*	0.7643	0.6995	0.0072	0.0003	0.3179	0.0672
Level						
Less 0.25	0.713±0.110	0.824±0.100	1.083±0.158^a^	1.403±0.270^a^	0.918±0.133	1.057±0.093
Requirement	0.775±0.112	0.875±0.056	0.705±0.093^b^	0.937±0.094^b^	1.042±0.080	0.868±0.075
Plus 0.25	0.684±0.075	1.174±0.189	0.905±0.142^a^	0.728±0.054^b^	1.182±0.158	1.050±0.116
*P value*	0.8253	0.1058	0.0805	0.0018	0.3155	0.2617
Source x Level						
DL-Met						
Less 0.25	0.743±0.155	1.029±0.133^a^^b^	0.791±0.170^b^^c^	0.741±0.042^b^	0.610±0.127^b^	0.911±0.105
Requirement	0.790±0.158	0.965±0.046^a^^b^	0.771±0.168^c^	0.727±0.099^b^	1.153±0.077^a^	0.917±0.092
Plus 0.25	0.695±0.140	0.960±0.163^a^^b^	0.561±0.163^c^	0.730±0.069^b^	1.167±0.277^a^	0.849±0.063
DL-HMTBA						
Less 0.25	0.683±0.171	0.618±0.097^b^	1.374±0.219^a^	2.066±0.381^a^	1.227±0.156^a^	1.202±0.138
Requirement	0.760±0.174	0.785±0.092^b^	0.639±0.090^c^	1.146±0.109^b^	0.930±0.131^a^^b^	0.819±0.124
Plus 0.25	0.674±0.072	1.388±0.335^a^	1.249±0.123^a^^b^	0.726±0.090^b^	1.197±0.182^a^	1.251±0.198
*P value*	0.9908	0.0560	0.0332	0.0021	0.0549	0.1377

^a-c^Different letters in the same column indicate a statistical difference by test t with p<0.10.

^1^Each value represents the average of 6 repetitions. Mean±SEM.

Observing the effects of dietary levels of methionine, methionine deficiency resulted in higher expression of the MTRR gene (p = 0.0018) compared to other levels. There was also lower expression of the BHMT gene (p = 0.0805), at the level at which the methionine requirement was met.

**Fig 2:**
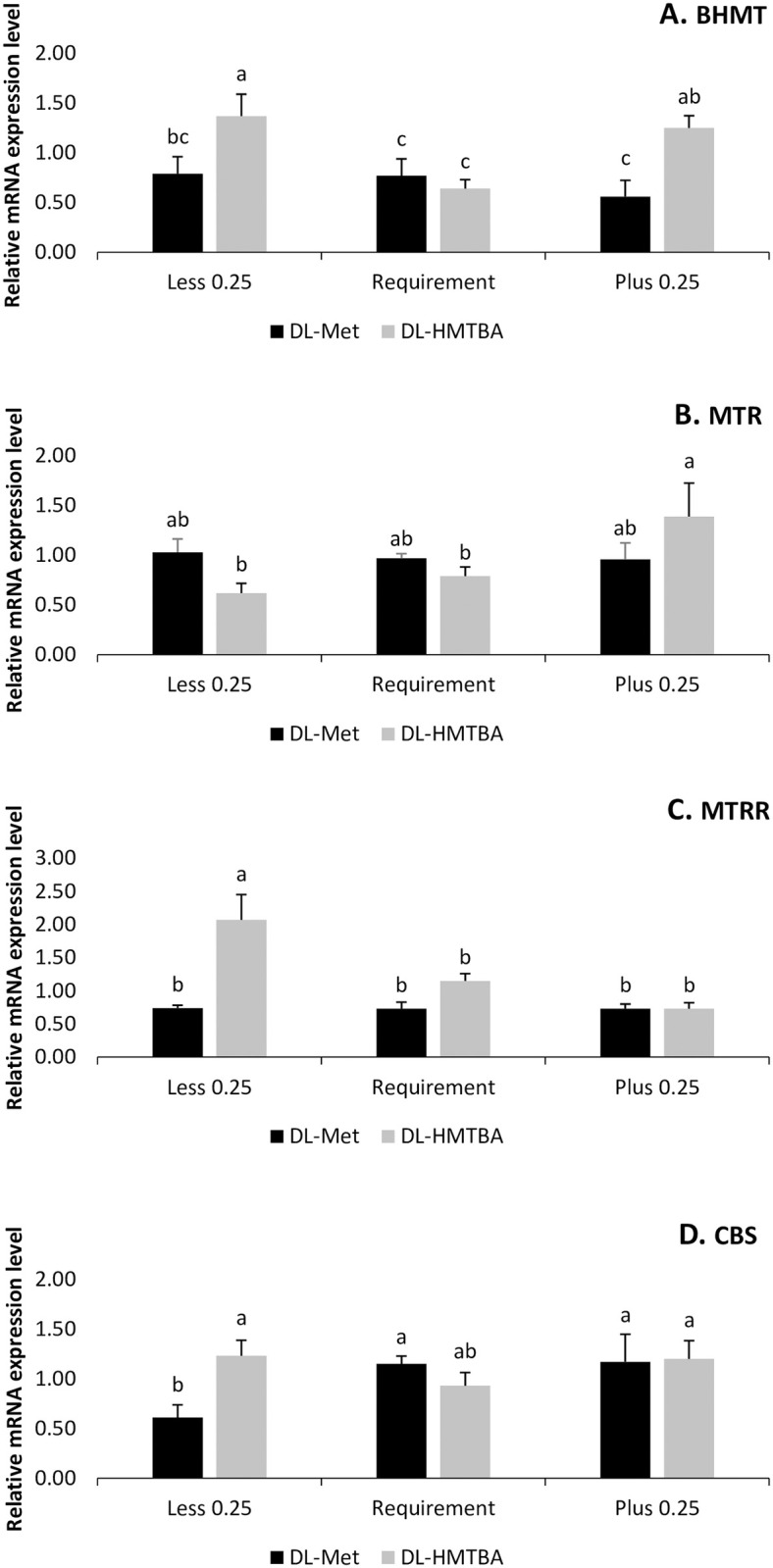
Graphic representation of the mRNA expression of (A.) betaine-homocysteine S-methyltransferase (BHMT), (B.) 5 methyltetrahydrofolate-homocysteine methyltransferase (MTR), (C.) 5-methyltetrahydrofolate-homocysteine methyltransferase reductase (MTRR) and (D.) cystathionine beta synthase (CBS) in the jejunum of broilers (mean±SEM; n = 6). ^a-c^Different letters indicate a statistical difference by test t with p<0.10.

There was an interaction effect between sources and levels of methionine for BHMT (p = 0.0332), MTRR (p = 0.0021), MTR (p = 0.0560) and CBS (p = 0.0549) genes. The highest expression of the MTR gene occurred in the condition of supplementation with DL-HMTBA in excess, and the lowest expression was for supplementation with DL-HMTBA in deficient and requirement levels of methionine (Fig 2).

For the BHMT gene, there was greater expression of the gene at the deficiency and excess levels of methionine with the use of DL-HMTBA, and lower gene expression occurred with the use of DL-Met at all levels tested and also the requirement level with DL-HMTBA (Fig 2). Using this source at the deficient level also increased MTRR expression compared to the other levels tested with DL-HMTBA and DL-Met (Fig 2).

**Fig 3:**
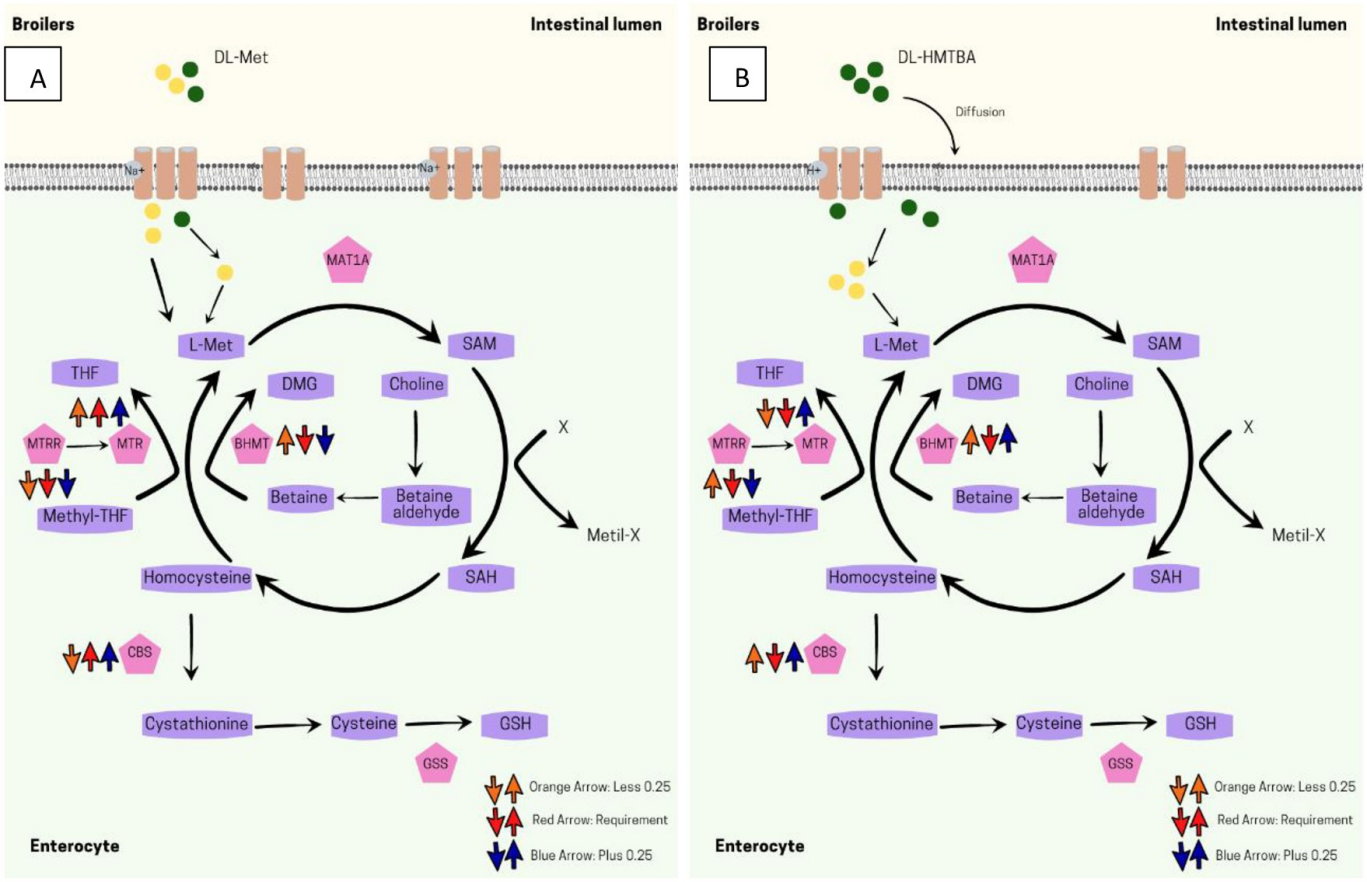
Integrated model that describes the mechanism by which (A.) DL-methionine and (B.) DL-HMTBA alter expressions of genes related to methionine metabolism in jejunal enterocytes of broilers. The studied genes are represented in pink pentagons. Up arrows indicate: *up-regulation*, down arrows indicate: *down-regulation*. MAT1: Methionine Adenosyltransferase 1; BHMT: Betaine-homocysteine S-methyltransferase; MTR: 5 methyltetrahydrofolate-homocysteine methyltransferase; MTRR: 5-methyltetrahydrofolate-homocysteine methyltransferase reductase; CBS: Cystathionine beta synthase; GSS: Gluthathione synthetase; SAM: S-Adenosyl methionine; SAH: S-Adenosyl-homocysteine; DMG: N, N-Dimethyglycine; THF: Tetrahydrofolate; GSH: Glutathione.

The use of DL-Met at the deficient level of methionine resulted in lower CBS gene expression when compared to the requirement group with DL-HMTBA. The other levels increased the expression of this gene in the jejunum of broilers (Fig 2). The gene expressions most affected by nutritional levels and sources of methionine were those of genes related to the remethylation process, which consists of the conversion of homocysteine to methionine, either via the tetrahydrofolate pathway or via the choline-betaine pathway. Furthermore, the conversion of homocysteine to cysteine was also affected by the sources of variation in this study (Fig 3).

Data on feed intake, weight gain, and feed conversion are shown in Table 5. The sources of methionine used in the study did not influence the feed intake of the birds, but the dietary levels of methionine had an effect (p = 0.0214); birds receiving a methionine-deficient diet increased their feed intake.

**Table 5:** Effect of increasing levels of methionine+cystine and replacement of DL-Met by DL-HMTBA in the diet on the performance of broilers from 8 to 42 days of age.

Main effects^1^	Feed intake (g/bird)	Weight gain (g/bird)	Feed conversion (g/g/bird)
Source			
DL-Met	4715.150±27.580	2994.950±35.484^a^	1.578±0.021^b^
DL-HMTBA	4708.380±26.411	2942.530±27.854^b^	1.602±0.018^a^
*P value*	0.8460	0.0117	0.0170
Level			
Less 0.25	4781.230±31.695^a^	2814.030±15.111^c^	1.699±0.008^a^
Requirement	4659.780±31.873^b^	2995.170±17.091^b^	1.556±0.010^b^
Plus 0.25	4694.280±26.184^b^	3097.020±25.480^a^	1.516±0.007^c^
*P value*	0.0214	< .0001	< .0001
Source x Level			
DL-Met			
Less 0.25	4791.861±44.446	2827.951±27.700^c^	1.694±0.011
Requirement	4621.970±41.807	2995.826±15.132^b^	1.543±0.018
Plus 0.25	4731.614±34.933	3161.065±27.394^a^	1.497±0.010
DL-HMTBA			
Less 0.25	4770.602±48.984	2800.118±12.649^c^	1.703±0.014
Requirement	4697.598±46.370	2994.509±32.497^b^	1.568±0.007
Plus 0.25	4656.942±35.195	3032.965±21.572^b^	1.535±0.003
*P value*	0.2142	0.0309	0.4664

^a-c^Different letters in the same column indicate a statistical difference by test t with p<0.05.

^1^Each value represents the average of 6 repetitions. Mean±SEM.

However, despite the higher feed intake, birds treated with the methionine-deficient diet were unable to convert the high feed intake into live weight gain and had the lowest weight gain among the three dietary levels (p<0.0001), and with that, the worst feed conversion (p<0.0001). Birds treated with the diet with excess methionine showed the highest weight gain and lowest feed conversion (p<0.0001).

Methionine sources significantly influence weight gain (p = 0.0117). DL-Met promoted greater weight gain and, consequently, lower feed conversion (p = 0.0170), since the feed intake of the birds was the same and was not influenced by the sources of methionine.

There was an interaction effect between sources and levels of methionine, for weight gain variable. Birds that received diets supplemented with DL-Met and dietary excess of methionine showed greater weight gain (p = 0.0309). However, this variation was not enough to change the feed conversion; the latter did not show an interaction effect between the factors studied.

## Discussion

The results visualized in the performance of the birds are due to the biochemical differences of the sources and the different levels of methionine, which influenced the metabolism of methionine in different ways. The MAT1 enzyme has the function of catalyzing the conversion of L-Met into SAM. All treatments were able to stimulate the MAT1 expression in the same way, with no differences among them. Other authors also did not find differences for the expression of MAT1 in the liver of broilers fed with DL-Met or DL-HMTBA, in different levels of methionine [6].

On the other hand, DL-Met and DL-HMTBA activated both remethylation pathways, mainly in conditions of methionine deficiency. While evaluating the duodenum of broilers, some authors observed that when the birds were fed with DL-Met, the choline-betaine pathway was more stimulated, with greater expression of BHMT, while DL-HMTBA stimulated the tetrahydrofolate pathway more, with greater expression of the MTR and MTRR genes [12].

However, in this study, we observed that DL-HMTBA stimulated the tetrahydrofolate pathway more than the choline-betaine pathway. Authors demonstrated that the tetrahydrofolate pathway is the main methionine remethylation pathway in broilers, while the choline-betaine pathway is a secondary pathway and is used on a smaller scale [13].

Due to its non-amino acidic nature, the use of DL-HMTBA as a source of methionine may cause the bird’s body to release signs of methionine deficiency [6]. Methionine deficiency signaling increased utilization of the methionine regeneration secondary pathway may be related to the higher expression of BHMT with the use of DL-HMTBA.

SAM is an allosteric regulator of the methionine cycle and the folate cycle. Low levels of SAM lead to increased activity of the tetrahydrofolate pathway, increasing homocysteine remethylation and consequently restoring normal methionine levels [14]. In situations of methionine deficiency, as observed in this study, in the treatment with methionine deficiency, there was an increase in MTRR expression, an effect which is observed more prominently in birds fed with DL-HMTBA.

However, the same effect was not observed for the MTR gene, in which there was greater expression in birds fed DL-HMTBA in methionine excess condition, which reinforces the hypothesis that DL-HMTBA causes signs of methionine deficiency. SAM is also a regulator of transsulfuration. When at low levels, transsulfuration is inhibited, for homocysteine conservation for remethylation [12].

However, some authors have found that low levels of sulfur amino acids stimulate the transsulfuration pathway to meet cystine requirements [15]. In this study, it was observed that DL-HMTBA stimulated transsulfuration at all levels, while DL-Met decreased CBS gene expression when supplemented at methionine-deficient levels.

Other authors found similar effects, higher gene expression of CBS in the liver, in the pectoralis major and in the gastrocnemius of chickens fed with DL-HMTBA, except for the duodenum, which showed a higher expression of CBS for broilers fed with DL-Met [12]. GSH biosynthesis occurs from three amino acids, namely cysteine, glutamic acid and glycine, which also requires the action of the GSS enzyme [3].

GSH is related to antioxidant activity, reducing the effects of free radicals (peroxides) from the metabolism or external sources [16]. Stress factors can lead to the production of free radicals, and consequently increase GSH levels, to mitigate their deleterious effects. Methionine deficiency can be a stressor leading to increased GSH production [12].

Some studies evaluating supplementation with DL-Met and DL-HMTBA in broilers concluded that DL-HMTBA promotes improved antioxidant activity due to lower lipid peroxidation and the best reduced-GSH:total-GSH ratio [17, 18]. Authors concluded that there are no gains for antioxidant activity of chickens fed with DL-HMTBA [16]. In this study, DL-HMTBA promoted greater expression of the GSH gene, reiterating the possibility that this source promotes signs of methionine deficiency in broilers.

For some authors, both sources are capable of providing methionine for the metabolism of growing broilers (1 to 25 days), with no differences in the performance of the birds; however, the authors found that methionine deficiency leads to performance losses, regardless of the source used [19]. Others report that in tropical conditions there is also no difference for the use of DL-Met and DL-HMTBA in broilers (1 to 35 days) [20].

However, researchers working with broilers (1 to 35 days old) found that DL-HMTBA, replacing 100% DL-Met, resulted in less weight gain and worse feed conversion, similar to the results found in the present study [21]. Methionine supplementation has a positive effect on muscle protein synthesis, due to its action on the expression of genes related to growth, such as IGF1 and GH [22].

The addition of methionine to broiler diets also improves amino acid balance, promoting growth and protein synthesis, reducing fat synthesis, and improving feed conversion [7]. In fact, in our study, increasing levels of methionine in the diet increased weight gain and improved feed conversion in the birds.

Other researchers found similar results with broilers (1 to 42 days old), in which methionine-deficient diets worsened the performance of birds, but did not show major differences for treatments with methionine supply in requirement and in excess levels [23]. In the present study, excess methionine led to better weight gain when birds were fed DL-Met, which may be related to the absorption of these sources.

In the jejunum of chickens, DL-Met is absorbed by specific transporters of the b^0,+^ and L (sodium independent) systems, and of the y^+^, B and A (sodium dependent) systems, which act against the concentration gradient; while DL-HMTBA is absorbed mainly by diffusion, depending on the concentration gradient in the jejunum of chickens, saturation may occur [8, 24].

In a study with broilers (1 to 28 days), some authors observed that above the requirement levels of DL-HMTBA promotes superior results in the performance of birds compared to DL-Met [25]. This difference would be associated with the absorption and/or metabolic rate of DL-HMTBA, which promotes better growth responses at levels above the recommended level for sulfur amino acids. The opposite is also valid: DL-HMTBA worsens the performance of birds in situations of methionine deficiency.

## Conclusion

Supplementation with DL-HMTBA leads to a slight deterioration in the performance of broilers, compared to supplementation with DL-Met. Both methionine sources studied in this work stimulated the methionine remethylation pathways, but DL-HMTBA stimulated the tetrahydrofolate pathway more, with greater expression of the BHMT and MTRR genes. In addition, birds fed DL-HMTBA showed signs of methionine deficiency.
